# Optical Characterization of the Interband Cascade LWIR Detectors with Type-II InAs/InAsSb Superlattice Absorber

**DOI:** 10.3390/nano14171393

**Published:** 2024-08-26

**Authors:** Krzysztof Murawski, Kinga Majkowycz, Małgorzata Kopytko, Tetiana Manyk, Karol Dąbrowski, Bartłomiej Seredyński, Łukasz Kubiszyn, Piotr Martyniuk

**Affiliations:** 1 Institute of Applied Physics, Military University of Technology, 2 Kaliskiego Street, 00-908 Warsaw, Poland; kinga.majkowycz@wat.edu.pl (K.M.); malgozata.kopytko@wat.edu.pl (M.K.); tetjana.manyk@wat.edu.pl (T.M.); piotr.martyniuk@wat.edu.pl (P.M.); 2 VIGO Photonics S.A., 129/133 Poznańska Street, 05-850 Ożarów Mazowiecki, Poland; kdabrowski@vigo.com.pl (K.D.); bseredynski@vigo.com.pl (B.S.); lkubiszyn@vigo.com.pl (Ł.K.)

**Keywords:** InAs/InAsSb, infrared detectors, defect states, photoluminescence, spectral response, cascade infrared detectors

## Abstract

The long-wave infrared (LWIR) interband cascade detector with type-II superlattices (T2SLs) and a gallium-free (“Ga-free”) InAs/InAsSb (x = 0.39) absorber was characterized by photoluminescence (PL) and spectral response (SR) methods. Heterostructures were grown by molecular beam epitaxy (MBE) on a GaAs substrate (001) orientation. The crystallographic quality was confirmed by high-resolution X-Ray diffraction (HRXRD). Two independent methods, combined with theoretical calculations, were able to determine the transitions between the superlattice minibands. Moreover, transitions from the trap states were determined. Studies of the PL intensity as a function of the excitation laser power allowed the identification of optical transitions. The determined effective energy gap (E_g_) of the tested absorber layer was 116 meV at 300 K. The transition from the first light hole miniband to the first electron miniband was red-shifted by 76 meV. The detected defects’ energy states were constant versus temperature. Their values were 85 meV and 112 meV, respectively. Moreover, two additional transitions from acceptor levels in cryogenic temperature were determined by being shifted from blue to E_g_ by 6 meV and 16 meV, respectively.

## 1. Introduction

Infrared (IR) sensors are widely used in many fields, including medicine, telecommunications, astronomy and the military. The devices are characterized by high detection parameters.

Their fabrication difficulties (especially in terms of long-wavelength infrared detectors—LWIR) have motivated the global efforts of research institutes and industrial companies dealing with IR detection to find new solutions. Currently, the dominant technology for IR detectors on the market is HgCdTe (MCT). However, fabrication difficulties have given the opportunity explore to A^III^B^V^ materials technology, especially InAs/InAsSb and InAs/GaSb type-II superlattices (T2SLs).

T2SLs, especially gallium (“Ga-free”) InAs/InAsSb, have many advantages as detection devices. Detectors based on this technology can operate in a wide range of the electromagnetic spectrum [1,2,3,4]. Moreover, the technology enables operation at high temperatures (high-operating temperature—HOT). The InAs/InAsSb T2SLs advantage is a longer minority carrier lifetime than InAs/GaSb. This is due to the suppression of the Auger recombination [5,6]. The available papers report a lifetime of several hundred ns in LWIR [7,8].

On the other hand, the LWIR InAs/InAsSb T2SLs’ disadvantages are weaker absorption and more difficult hole transport as compared to its InAs/GaSb counterpart. Therefore, for InAs/InAsSb T2SLs, a longer superlattice period is necessary to get the same band gap as for the InAs/GaSb T2SLs. The literature data show that the absorption coefficients for InAs/InAsSb T2SLs with a band gap of ~0.1 eV (corresponding to the cutoff wavelength λ_cutoff_ = 10–12 µm) are approximately half of those for InAs/GaSb ones [9]. Moreover, this results in larger growth-direction hole conductivity effective masses, which in turn lead to lower vertical hole mobility and shorter diffusion length [10,11,12,13,14]. A longer superlattice period may influence the presence of defect states. The presence of additional recombination centres increases the Shockley–Read–Hall (SRH) recombination. The growth of epitaxial layers free from structural defects is a continuous technological challenge.

Some information on the location of the trap levels in the energy gap has been previously reported, but the origin of the native point defects (NPDs) has been found to be still unknown. Particularly important is the work by S. Krishnamurthy and Yu Zhi Gang: “Green’s function-based defect identification in InAs-InAs_1-x_Sb_x_ strained layer superlattices” [15]. It shows possible NPDs typical for the ternary compound InAsSb and InAs. The NPDs in the InAs in both regions are V_In_, As_In_, V_As_ and, In_As_. Additionally, the V_Sb_, In_Sb_ and Sb_In_ are in the InAsSb region, respectively. Research on defective states provides the necessary knowledge for designing and producing ready-made, high-quality detection devices. 

An interband cascade infrared photodetector (ICIP) T2SL seems to be a solution to some issues. The use of a cascade structure gives a high total absorption while mitigating the limitations in carrier extraction due to the finite diffusion length [16,17,18]. Additionally, it reduces the overall noise and increases the correct detection.

This article presents the results of two measurement techniques: spectral response (SR) and photoluminescence (PL). The first one allowed for determining the levels between minibands in the active layer of LWIR ICIP with the T2SLs “Ga-free” InAs/InAsSb (x = 0.39) absorber. The second one, in addition to confirming the results, allowed us to isolate active optical transitions within the band gap. The interpretation of the results was additionally carried out using the PL intensity as a function of the excitation power. The obtained results were compared with the current state of knowledge available in the literature. Moreover, the crystallographic quality was confirmed by high-resolution X-Ray diffraction (HRXRD).

## 2. Materials and Methods

**Figure 1:**
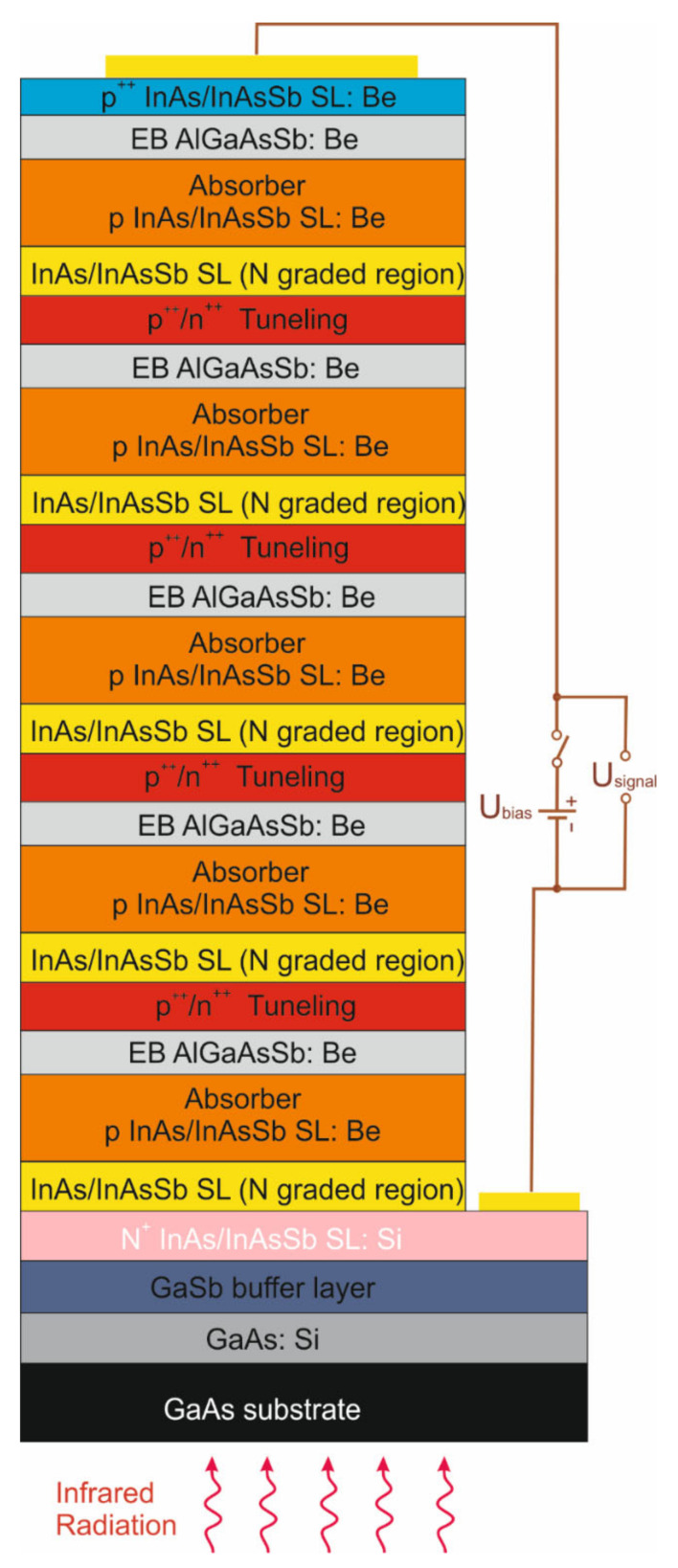
LWIR InAs/InAs_1-x_Sb_x_ T2SLs multi-junction cascade detector.

In our experiment, the devices were grown by a RIBER Compact 21-DZ solid source MBE system on the GaAs (001) substrate. The GaAs substrate get the possibility of an immersion lens formation—the monolithic technology developed at VIGO Photonics S.A. This solution makes it possible to increase the normalized detectivity of the detector. The 250 nm GaAs smoothing layer was deposited and followed by the deposition of the GaSb buffer layer. Next, a 1 µm heavily doped wide-gap N^+^ contact layer, an active layer and a 0.1 μm heavily doped wide-gap p^+^ bottom layer serving as the hole contact were deposited. The analyzed detectors consisted of five stages (five active layers connected in series). The single stage consisted of a 0.1 μm graded (by layer thickness) InAs/InAsSb N-type layer 0.59 µm thick, a 9.83 nm T2SLs period InAs/InAsSb x_Sb_ = 0.39 absorber, a 0.195 μm AlGaAsSb:Be bulk barrier (EB) and a 0.16 μm InAs/InAsSb p^+^ layer serving as the hole contact. The stages were connected by the same design of InAs/InAsSb with heavily doped p^++^/n^++^ tunnel junctions. The absorber was optimized to operate up to 10.6 μm at 300 K. Figure 1 shows the architecture of the N-stage cascade heterostructures. The detailed growth technique and procedure is described in Refs. [19,20].

For the SR measurements, the mesa sample was processed using standard processing such as photolithography, chemical etching, and metallization. The device was mounted on the TO8 stand, bonded and placed in a helium cryostat.

**Figure 2:**
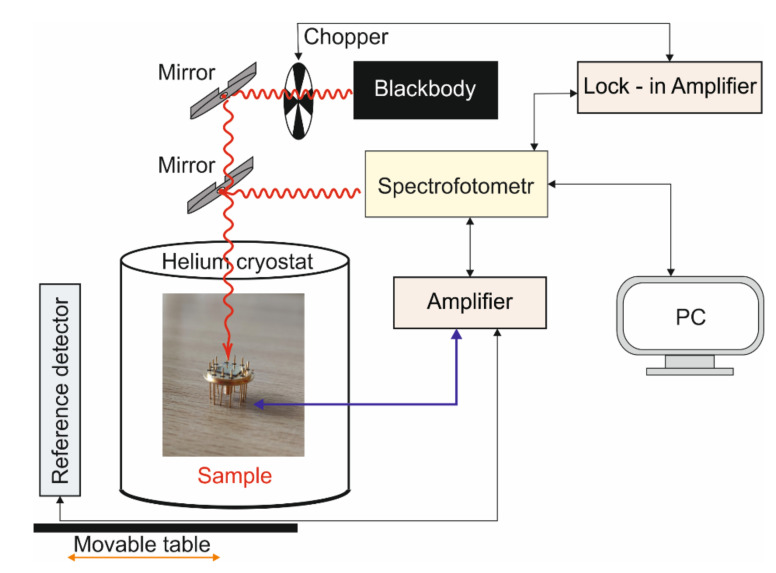
SR measurement system.

The SR measurements were performed using the FTIR type Spectrum 2000 spectrophotometer with a HTB-35D-1200 blackbody at a temperature of 1000 K and a DLTGS LEONARDO pyroelectric reference detector. The sample and reference detector were on the movable table. All characteristics were measured for a bias of −0.3 V and temperatures within the range of 300 K to 70 K. The electrical area was 7.85 × 10^−5^ cm^2^. The diagram of the measurement system is shown in Figure 2.

**Figure 3:**
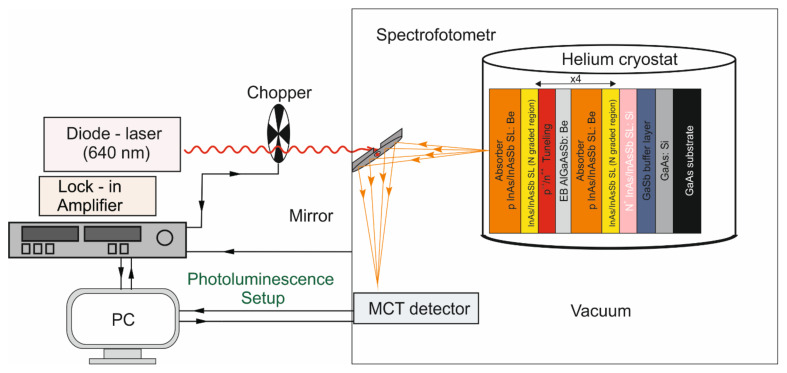
PL measurement system.

PL is a method for analyzing transitions between bands and shallow impurity states. The line width of the PL spectrum and the peak energy provide information about the concentration of the dopants and their types, material quality, and composition fluctuations. For PL measurements, all structures were chemically etched into the absorber layer by H_3_PO_4_ + C_2_H_8_O_7_ + H_2_O_2_ + H_2_O (molar ratio: 1:1:4:16). The PL measurement system was shown in Figure 3. The characteristics were measured in a vacuum using the FTIR Bruker Vertex 70v spectrometer in the step-scan mode [21], the liquid-nitrogen cooled MCT DS315 detector, and a lock-in amplifier. As a source, the 640 nm line, mechanically chopped with a frequency of a 10 kHz laser, was used. PL spectra were collected for a temperature range from 300 K to 20 K and an excitation power from 200 mW to 10 mW. The diameter of the laser beam was about 1.5 mm.

## 3. Results

**Figure 4:**
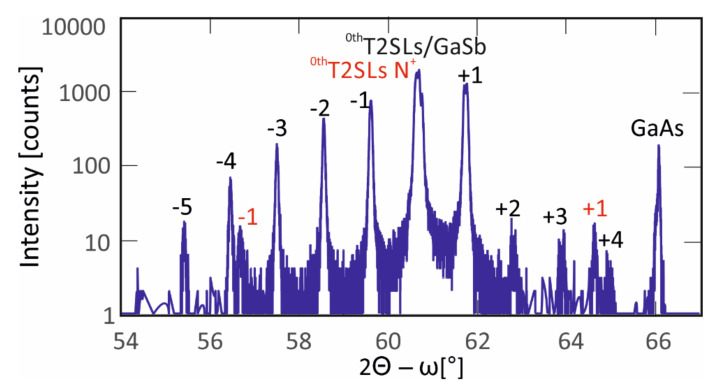
HRXRD LWIR InAs/InAs_1-x_Sb_x_ T2SLs (x = 0.39) spectra at 300 K.

The detection structures must be characterized by the highest quality and accuracy of growth. HRXRD in 2Θ-ω direction measurements were performed to confirm the crystallographic quality. Results are presented in Figure 4. The 66.05° peak corresponds to the GaAs substrate. The black satellites correspond to the absorber and p^++^/n^++^ tunneling layers. They were made of InAs/InAsSb T2SLs for the same molar compositions and constituent layer thickness as the active layers. The red ones correspond to the N^+^ and p^+^ T2SLs. The 0^th^ order satellite FWHM~190 arcsec confirms good lattice matching among the InAs/InAsSb T2SLs, the GaSb buffer, and the bulk electron barrier. The period was determined based on the space between the satellites.

**Figure 5:**
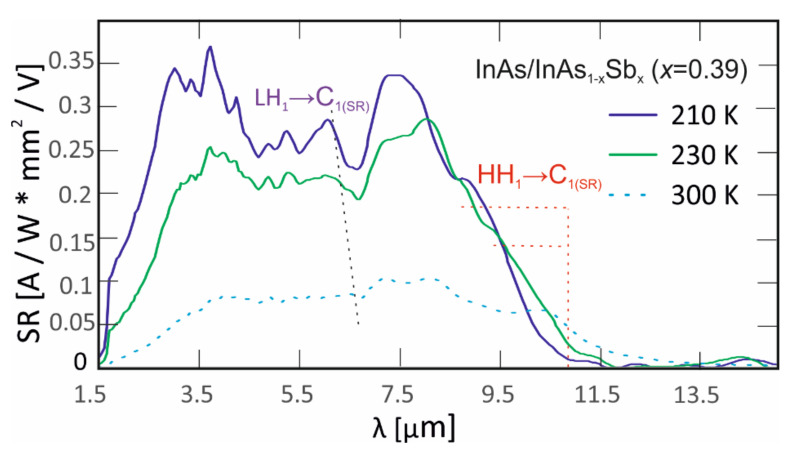
SR spectra LWIR InAs/InAs_1-x_Sb_x_ T2SLs (x = 0.39) at high temperatures.

Figure 5 shows the SR versus wavelength from 210 K to 300 K. The fundamental transition (E_g_) of InAs/InAsSb T2SL is determined as an energy between the highest heavy-hole miniband (HH_1_) and the lowest electron miniband (C_1_) in the centre of the Brillouin zone [22]. The E_g_ in this case, is equivalent to the point at which the detector response drops to 50% of the peak value [23]. Apart from the HH_1_→C_1_ transition, there is also a visible transition from the first light-hole miniband to the first electron miniband (LH_1_→C_1_). The position of the minibands depends on the thicknesses of the component layers, the molar composition of InAsSb, and the shift of the bands of the individual layers. The HH_1_→C_1_ position varied from 9.2 μm to 10.6 μm for the temperature range from 210 K to 300 K. LH_1_→C_1_ was shifted by approximately 4 μm. However, to clearly determine the value, PL measurements were carried out.

**Figure 6:**
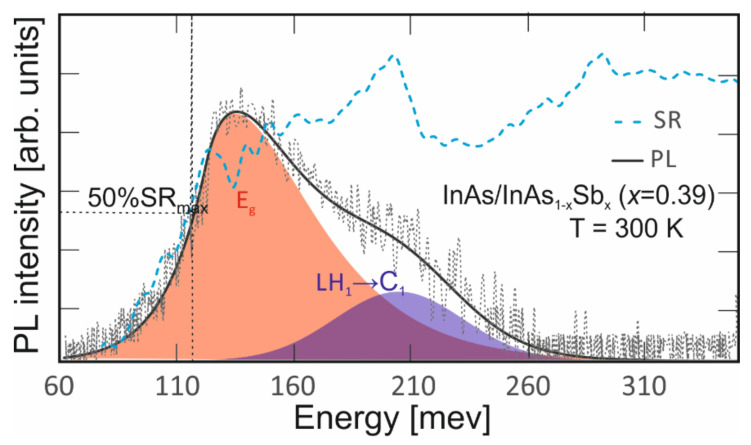
PL and SR LWIR InAs/InAs_1-x_Sb_x_ T2SLs (x = 0.39) spectra at 300 K.

Spectra for both methods at 300 K are shown in Figure 6. The band-to-band transitions from the PL measurement are proportional to the product of the density of states and the Fermi–Dirac distribution function [24]. Similar HH_1_→C_1_ and LH_1_→C_1_ transition values were obtained for both measurement methods (for HH_1_→C_1_ 116 meV, and LH_1_→C_1_ 190 meV, respectively). Approximately 76 meV HH_1_→C_1_ to LH_1_→C_1_ transitions were confirmed by theoretical calculations using APSYS simulations. 

**Figure 7:**
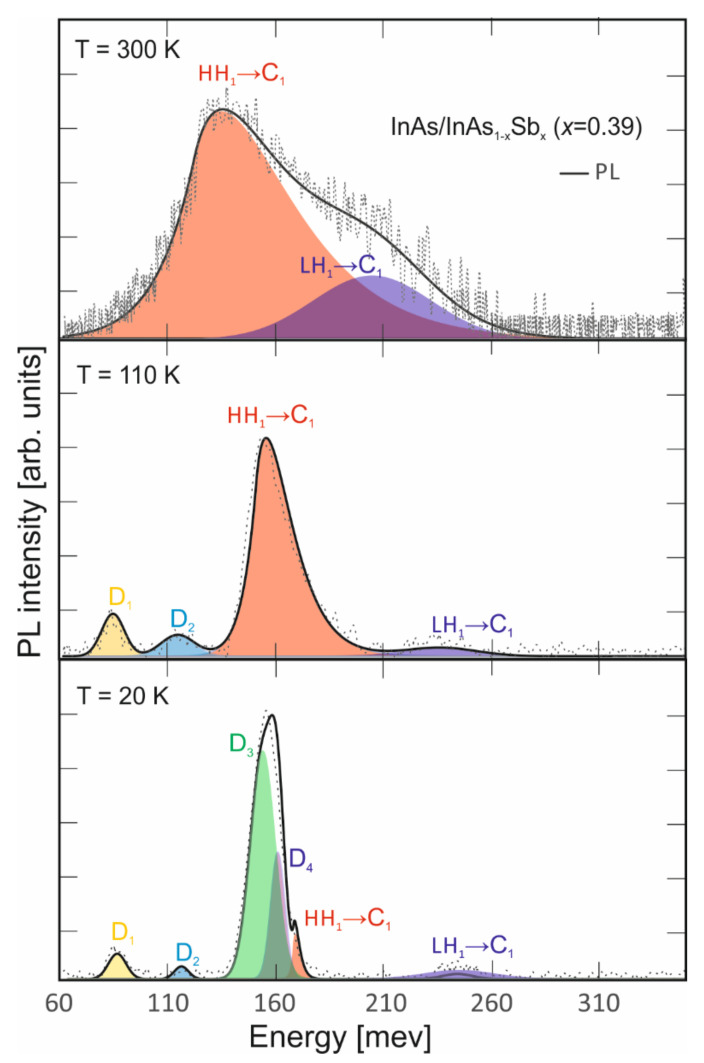
PL LWIR InAs/InAs_1-x_Sb_x_ T2SLs (x = 0.39) spectra at temperatures 20 K, 110 K and 300 K.

The PL results at 300 K, 110 K and 20 K presenting dynamic change optical transitions versus temperature are shown in Figure 7. At the PL for 300 K, the HH_1_→C_1_ and LH_1_→C_1_ transitions described above were observed. As the temperature decreased, additional transitions were observed. For the 110 K, two additional transitions were observed: D_1_ and D_2_, respectively. The position of these transitions was about 85 meV and 112 meV, respectively. The positions were determined by the Gaussian density of states. HH_1_→C_1_ and LH_1_→C_1_ transition were observed at 131 meV and 209 meV, respectively. 

At 20 K, PL spectra was dominated by six transitions: HH_1_→C_1_, LH_1_→C_1_, D_1_, D_2,_ D_3_ and D_4_. The D_3_ intensity clearly dominated, but LH_1_→C_1_ was barely visible. The HH_1_→C_1_ and LH_1_→C_1_ energies were about 167 meV and 239 meV, respectively. Moreover, it could be seen that these bands were slightly closer. The D_3_ and D_4_ positions were shifted to E_g_ by 6 meV and 16 meV, respectively. Furthermore, the D_1_ and D_2_ positions were constant versus the temperature. 

**Figure 8:**
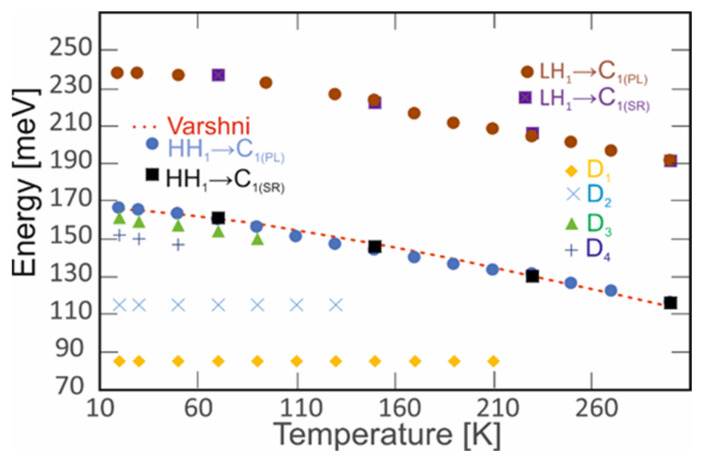
The temperature dependence of the energy peaks for the LWIR InAs/InAs_1-x_Sb_x_ T2SLs (x = 0.39). The dashed red curve presented the Varshni fitting to the HH_1_→C_1_, experimental rosulates.

The transitions’ temperature dependence analysis is presented in Figure 8. At high temperatures, the PL spectrum originated from a free-carrier emission. It was dominated by the transitions between the minibands up to 210 K. Below 210 K, an additional transition with an energy of 85 meV was observed. The next transition was observed (D_2_) below 130 K. The D_1_ and D_2_ positions were constant over the entire temperature range. Most likely, those transitions originate from the defect level to the valence band. The D_3_ and D_4_ transitions were observed only at cryogenic temperatures for temperatures below 90 K and 50 K. Their energies varied from 150 meV to 161 meV and from 147 meV to 152 meV, respectively. The LH_1_→C_1_ transition energy varied from 190 meV to 239 meV. Moreover, the LH_1_→C_1_ PL intensity disappeared as the temperature decreased. The HH_1_→C_1_ transition changed its value from 116 meV to 167 meV. 

As can be seen, the results for transitions between minibands obtained by both methods are similar. The origins of all transitions are discussed below. 

The analysis of the energy gap dependence on the temperature was compared to the empirical expression reported by Varshni [25]:

(1) $$E_{g}\left(T\right)=E_{0}-{\alpha_{0}T}^{2}/\left(T+\beta \right)$$

where *E_g_*_0_, $\alpha_{0}$ and β are the parameters for fitting the curve to the experimental points.

The following fitting parameters were obtained: *E_g_*_0_ = 167 meV, $\alpha_{0}$ = 0.29 meV/K and β=200K, respectively. Moreover, the origins of all PL peaks were analyzed as functions of the excitation power.

**Figure 9:**
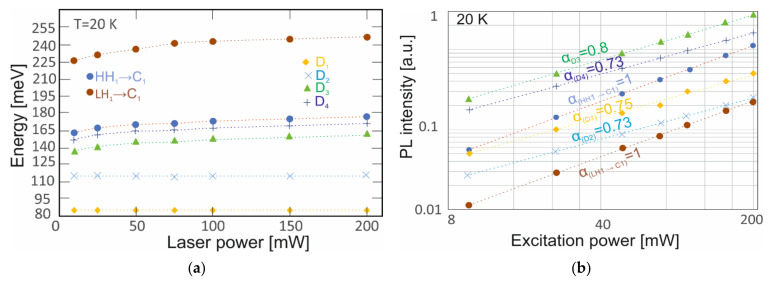
(**a**,**b**) PL intensity versus excitation power for the LWIR InAs/InAs_1-x_Sb_x_ T2SLs (x = 0.39) at 20 K.

Figure 9a shows the position of the emission peaks’ dependence on the excitation power at 20 K. The largest changes were observed for the transition LH_1_→C_1_. It varied accordingly from 243 meV to 226 meV with an excitation of 200 mW to 10 mW. Moreover, the HH_1_→C_1_, D_3_ and D_4_ transitions energies shifted to blue at about 3 meV. The energies D_1_ and D_2_ were constant, which is typical for deep-level trap transitions [26].

The PL intensity as a function of excitation power allowed us to identify the origin of the emission peaks [26,27,28,29,30,31]:

(2) $$I_{PL}=P^{\alpha }$$

where $I_{PL}$ is the PL peak intensity for the given excitation power P, and α indicates the transition type [26,27,28]. The α = 1 corresponds to the band-to-band transitions, while α < 1 corresponds to the shallow- or deep-defect transitions. The results are shown in Figure 9b. For HH_1_→C_1_ and LH_1_→C_1_ transitions, the α corresponds to an excitonic recombination. The α of D_1,_ D_2_, D_3_ and D_4_ PL peaks assume 0.75, 0.73, 0.8 and 0.73, respectively. The D_1_ and D_2_ transitions are classified as defect-bound excitons, while D_3_ and D_4_ are classified as acceptor-bound excitons.

**Figure 10:**
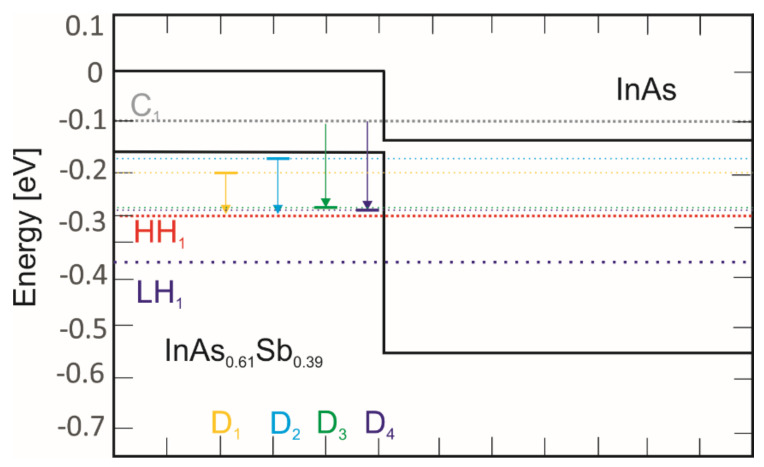
LWIR InAs/InAs_1-x_Sb_x_ T2SLs (x = 0.39) band diagram at 20 K. The solid black lines represent the conduction band (CB) and valence band (VB) of bulk InAs and InAsSb alloys.

Figure 10 shows the LWIR InAs/InAs_1-x_Sb_x_ T2SLs (x = 0.39) band diagram at 20 K. The solid black lines represent the conduction band (CB) and valence band (VB) of the bulk InAs and InAsSb alloys. The dashed lines represent sub-band energy levels and the defect levels that were determined.

The experimentally extracted defect level positions were compared with those reported for bulk InAs and InAsSb (x_Sb_ = 0.34) [15].

## 4. Discussion

This paper presents optical measurements by FTIR to investigate the properties of the LWIR HOT T2SLs InAs/InAsSb cascade detectors. The SR measurements show high results for the HOT condition [17,18,32]. Additionally, HRXRD measurements confirm the high crystallographic quality of the tested device. The method of determining the transitions between minibands using APSYS is presented in many literature reports [33,34]. The shift of 76 meV with respect to E_g_, obtained experimentally, seems to be confirmed using the theoretical model. However, interesting results from the PL measurements are presented. In addition to the transition between minibands, they show additional transitions at lower temperatures. The determination of E_g_ based on the optical measurements obtained by one method is itself ambiguous. The approach proposed in this article, which shows the results obtained for two different measurement methods, seems to be correct. Moreover, the PL intensity versus the excitation power results for the remaining visible transitions provide comprehensive information about their types. The results for the D_1_ and D_2_ transitions are particularly interesting. The α PL peaks assume 0.75 and 0.73, respectively. The α values 0.75 and 0.73 in the literature are assigned to the transitions as a defect-bound exciton and acceptor-bound exciton. [26,35]. However, the lack of peak shift with the change in excitation energy is typical to transitions involving defect states [28]. Some sources attribute the transition from around the mid-energy gap to a defect associated with V_In_ [8]. Krishnamurthy et al. in “Green’s function-based defect identification in InAs/InAs_1-x_Sb_x_ strained layer superlattices” indicates that In-vacancies are even easier to form in both regions of the superlattice than in the separate InAs or InAsSb bulk alloys. This would suggest that D_1_ comes from V_In_. However, comparing the results to the theoretical NDP position of bulk InAs and InAsSb, D_1_ corresponds more closely to In_As_ at the InAsSb region [15]. Moreover, the D_2_ position is close to the V_In_ value within the InAsSb reported in the literature. On the other hand, the impact of individual identified defects on device properties and performance is not known. The presence of defect levels reduces the carriers’ lifetimes and increases dark currents in detection devices. However, the low density of these defects would not have such a large impact. Additional studies are needed to clarify this.

At cryogenic temperatures, two additional optical transitions are visible: D_3_ and D_4_. The α for D_3_ and D_4_ PL peaks equal 0.8 and 0.73, respectively. They are classified as acceptor-bound excitons [26].

## 5. Conclusions

The LWIR ICIP built of the T2SLs Ga-free InAs/InAsSb (x = 0.39) absorbers was characterized by PL and SR methods. Heterostructures were grown by MBE on a GaAs substrate (001) orientation. The crystallographic quality was confirmed by HRXRD. The results, combined with theoretical analysis, confirmed the location of HH_1_→C_1_ and LH_1_→C_1_ miniband transitions of the examined heterostructure. Additionally, transitions of In_As_ and V_In_ at the InAsSb region were located. The detected defects’ state energy was constant with temperature. Their values were 85 meV and 112 meV, respectively. Moreover, two additional transitions from acceptor levels in cryogenic temperature were extracted. Their energies were blue-shifted from E_g_ to 6 meV and 16 meV, respectively.

## Data Availability

Data are contained within the article.
